# 14‐Day Vonoprazan–Minocycline Dual Therapy Versus Bismuth‐Containing Quadruple Therapy for *Helicobacter pylori* Rescue Treatment: A Single‐Center Randomized Controlled Noninferiority Trial

**DOI:** 10.1111/hel.70153

**Published:** 2026-08-20

**Authors:** Tingting Yang, Ruijuan Xin, Yanhong Deng, Jun Liu, Xue Li, Xiaole Guo, Linke Ma, Xiaoming Su, Yanxia Liu, Chengdong Su, Qian Hao, Rui Mu, Xiaofei Li, Pengda Wang, Yanling Li, Xue Zhang, Qingrui Li, Yuting Wu, Shengjuan Hu

**Affiliations:** ^1^ The Third Clinical Medical College Ningxia Medical University Yinchuan China; ^2^ Department of Gastroenterology, People's Hospital of Ningxia Hui Autonomous Region Affiliated People's Hospital of Ningxia Medical University Yinchuan China

**Keywords:** dual therapy, *Helicobacter pylori*, minocycline, rescue treatment, vonoprazan

## Abstract

**Background:**

Rescue treatment for *Helicobacter pylori* infection remains challenging in regions with high antibiotic resistance. Although bismuth‐containing quadruple therapy is widely recommended as an empirical rescue regimen, its medication burden may impair tolerability and adherence. This trial evaluated the efficacy and safety of 14‐day vonoprazan–minocycline dual therapy compared with vonoprazan‐based bismuth‐containing quadruple therapy as empirical rescue treatment.

**Materials and Methods:**

This single‐center, prospective, open‐label, randomized, controlled, noninferiority trial enrolled 186 adults aged 18–70 years with at least one previous *H. pylori* eradication failure. Patients were randomly assigned (1:1) to receive 14‐day vonoprazan–minocycline dual therapy or vonoprazan‐, amoxicillin‐, minocycline‐, and bismuth‐containing quadruple therapy. Eradication was assessed by a ^13^C urea breath test 4 weeks after treatment. ITT, mITT, and PP analyses were performed, with a prespecified noninferiority margin of −10%.

**Results:**

Eradication rates in the dual therapy and quadruple therapy groups were 89.2% and 91.4% in the ITT analysis (difference, −2.2%; 95% CI, −10.5% to 6.2%; noninferiority *p* = 0.036), 90.2% and 92.4% in the mITT analysis (difference, −2.2%; 95% CI, −9.7% to 5.3%; noninferiority *p* = 0.030), and 91.2% and 93.4% in the PP analysis (difference, −2.2%; 95% CI, −9.4% to 5.0%; noninferiority *p* = 0.024). Noninferiority was demonstrated in the mITT and PP analyses, whereas the ITT result should be interpreted cautiously because the lower 95% CI bound crossed the margin. Adverse events were similar between treatment groups (11.8% vs. 15.1%; *χ* ^2^ = 0.416; *p* = 0.519), and adherence was high in both groups (97.8% vs. 97.8%; *p* = 1.000).

**Conclusions:**

14‐day vonoprazan–minocycline dual therapy achieved eradication rates comparable to those of bismuth‐containing quadruple therapy, with acceptable safety and high adherence as rescue treatment for *H. pylori* infection. This simplified regimen may be considered a potential empirical rescue option when susceptibility testing is not readily available, although the ITT noninferiority result warrants cautious interpretation.

## 1 Introduction

*Helicobacter pylori* ( *H. pylori* ) is a Gram‐negative pathogenic bacterium that primarily colonizes the gastric and duodenal mucosa and is closely associated with the development and progression of chronic gastritis, peptic ulcer disease, and gastric cancer [1, 2]. Epidemiological data from China indicate a substantial burden of *H. pylori* infection. In a study involving 10,735 households, the household‐level prevalence of *H. pylori* infection was reported to be as high as 71.21% [3, 4], whereas the individual‐level prevalence was 43.9% [5]. In 1994, the International Agency for Research on Cancer (IARC) classified *H. pylori* as a Group 1 carcinogen, and the Maastricht VI/Florence Consensus explicitly recommends eradication therapy for all infected individuals [6]. Therefore, improving the accessibility and effectiveness of eradication therapy is of considerable public health importance.

At present, *H. pylori* eradication therapy relies primarily on antibiotics such as amoxicillin, clarithromycin, levofloxacin, metronidazole, furazolidone, and tetracycline; however, the growing problem of antimicrobial resistance has substantially reduced treatment efficacy. Although bismuth‐containing quadruple therapy has been recommended as a first‐line regimen in Chinese consensus guidelines [7], nonstandard triple regimens, inappropriate antibiotic use, and inadequate treatment duration remain common in routine primary care practice. These shortcomings not only increase the likelihood of initial treatment failure and further exacerbate the burden of antimicrobial resistance but also create an increasingly urgent need for effective rescue therapy. According to a meta‐analysis of studies conducted in mainland China between 2000 and 2023 [3], the primary resistance rates of *H. pylori* to clarithromycin, metronidazole, and levofloxacin were approximately 30%–33%, 40%–97%, and 29%–37%, respectively, whereas the corresponding secondary resistance rates rose to 58%–89% after eradication failure. In contrast, both primary and secondary resistance rates to amoxicillin, tetracycline, and furazolidone have generally remained relatively low, ranging from < 5% to 15%. Consistent with these changing resistance patterns, the failure rate of first‐line eradication therapy in China has reached approximately 15%–25%, rendering rescue treatment a major contemporary clinical challenge. Moreover, patients requiring rescue eradication therapy often face more complex resistance profiles due to prior cross‐exposure to antibiotics, cumulative adverse effects, and reduced treatment adherence, all of which may limit the consistency and reproducibility of empiric rescue regimens [5].

The intensity of intragastric acid suppression is a key determinant of *H. pylori* eradication efficacy. As a potassium‐competitive acid blocker (P‐CAB), vonoprazan is characterized by a rapid onset of action and potent and sustained acid suppression, thereby maintaining intragastric pH within the range of 6.0–8.0 and providing a more stable microenvironment for antibiotic activity. In a large‐scale randomized, controlled trial, a 14‐day bismuth‐containing quadruple rescue regimen comprising vonoprazan, amoxicillin, and furazolidone (P‐CAB‐BQT), in which vonoprazan replaced rabeprazole, was shown to be not only noninferior but also superior to PPI‐BQT, with eradication rates of 91.9% versus 83.8% in the ITT analysis and 95.6% versus 86.3% in the PP analysis [8]. These findings suggest that more potent acid suppression may be a key factor contributing to improved rescue treatment outcomes.

With respect to antibiotic selection, tetracyclines have maintained relatively low resistance rates against *H. pylori* in mainland China (0.7%–8.2%), with limited cross‐resistance to commonly used first‐line agents, thereby making them potentially valuable options for rescue therapy. Minocycline, a tetracycline derivative, offers several potential advantages over tetracycline, including greater oral bioavailability and a longer half‐life, which allow once‐ or twice‐daily administration [9]. In addition, minocycline has a favorable safety profile, and resistance of *H. pylori* to minocycline appears to be uncommon, supporting its use as a potential alternative to tetracycline in bismuth‐containing quadruple rescue regimens. Under conditions of potent acid suppression and with the use of low‐resistance antibiotics, a dual regimen combining minocycline with vonoprazan may achieve acceptable eradication efficacy while reducing both antibiotic exposure and treatment complexity. In an open‐label randomized, controlled trial, 14‐day vonoprazan–minocycline dual therapy (VM) achieved eradication rates of 90.0% in the intention‐to‐treat (ITT) analysis and 96.4% in the per‐protocol (PP) analysis among treatment‐naive patients; among patients receiving rescue therapy, the corresponding eradication rates were 76.7% and 79.3%, respectively, with overall eradication rates of 83.3% in the ITT analysis and 87.7% in the PP analysis [10]. Furthermore, the addition of amoxicillin and bismuth to this dual regimen to form a more intensive combination strategy may theoretically improve eradication success, although such intensification may also increase treatment complexity and the burden of adverse events.

At present, high‐quality randomized controlled evidence regarding vonoprazan combined with minocycline as an empirical rescue regimen for *H. pylori* infection remains limited, and direct comparative data with vonoprazan‐based bismuth‐containing quadruple therapy are scarce. Against this background, we conducted a single‐center, randomized, controlled, noninferiority trial to compare the efficacy and safety of 14‐day vonoprazan–minocycline dual therapy with those of quadruple therapy containing vonoprazan, amoxicillin, minocycline, and colloidal bismuth pectin for rescue eradication of *H. pylori* infection. Treatment adherence and adverse events were also evaluated to further assess whether this simplified regimen may serve as a potential empirical rescue option in clinical settings where susceptibility testing is not readily available.

## 2 Materials and Methods

### 2.1 Study Design and Patients

This study was designed as a prospective, single‐center, open‐label, randomized, controlled, noninferiority trial. Patients were recruited from the *H. pylori* specialty clinic of Ningxia Hui Autonomous Region People's Hospital between February 2025 and February 2026. Eligible participants were those with confirmed *H. pylori* infection who had experienced at least one previous eradication failure.

The inclusion criteria were as follows: (1) age 18–70 years; (2) current *H. pylori* infection confirmed by a 13C‐urea breath test; (3) no use of P‐CABs, proton pump inhibitors, H2‐receptor antagonists, bismuth compounds, or other agents that might affect *H. pylori* activity within 2 weeks before enrollment; and (4) no use of antibiotics or Chinese herbal medicines with established antibacterial activity within 4 weeks before enrollment. The exclusion criteria were as follows: (1) prior use of a regimen containing vonoprazan, bismuth, minocycline, and amoxicillin in combination; (2) contraindications or a history of allergy to any study medication or to the drugs included in the control regimen; (3) a history of gastrointestinal bleeding, obstruction, perforation, malignancy, or other severe organic diseases; (4) severe cardiac, hepatic, or renal insufficiency or immunodeficiency; (5) psychiatric disorders, psychological conditions, or inability to cooperate with the investigators; (6) planned pregnancy, pregnancy, or lactation; and (7) concurrent participation in another clinical trial or refusal to provide written informed consent. All participants were enrolled voluntarily, had complete clinical data, agreed to undergo follow‐up assessment of treatment efficacy, and provided written informed consent.

The study protocol was approved by the Ethics Committee of Ningxia Hui Autonomous Region People's Hospital (Ethics No. [2025]‐LL‐008). This trial was conducted in accordance with the CONSORT statement for randomized, controlled trials and was registered with the Chinese Clinical Trial Registry under the title “Efficacy of Minocycline‐Containing Regimens in the Treatment of *Helicobacter pylori* Infection” (ChiCTR2600122365).

### 2.2 Treatment and Follow‐Up

The sample size for this study was estimated based on the per‐protocol (PP) eradication rates reported in previous studies of *H. pylori* rescue therapy, which ranged from approximately 91%–97% [11]. Assuming an eradication rate of 94.3% in both the experimental and control groups, a prespecified noninferiority margin of −10%, 80% power, a one‐sided α of 0.025, and a 1:1 allocation ratio, the required sample size was calculated using the formula for a noninferiority test for two independent proportions. The −10% margin was selected with reference to previous noninferiority studies in *H. pylori* rescue therapy and was considered clinically acceptable in this setting, given the potential benefits of the simplified dual regimen, including reduced medication burden, avoidance of bismuth and an additional antibiotic, improved treatment convenience, and acceptable safety and adherence [12]. On this basis, at least 84 patients were required in each group. After allowing for an anticipated 10% dropout rate, the final target sample size was set at 93 patients per group, yielding a total sample size of 186 participants. A total of 186 eligible patients were randomly assigned in a 1:1 ratio to either the vonoprazan–amoxicillin–minocycline–colloidal bismuth pectin quadruple therapy group (hereafter, the bismuth quadruple group) or the vonoprazan–minocycline dual therapy group (hereafter, the minocycline dual group). The randomization sequence was generated by an independent statistician using a computer‐generated randomization program. Treatment allocation was concealed in consecutively numbered, opaque, sealed envelopes and was maintained by an independent researcher, thereby minimizing selection bias. Owing to differences in regimen composition and medication burden between the two groups, an open‐label design was adopted, in which neither participants nor investigators were blinded.

Before treatment initiation, all patients received detailed instructions from the study staff regarding medication administration, potential adverse events, and the importance of strict adherence to the prescribed regimen. The treatment regimen for the bismuth quadruple group was as follows: vonoprazan 20 mg (Takeda Tianjin Pharmaceutical Co. Ltd.) administered orally twice daily, 30 min before meals; colloidal bismuth pectin 300 mg (Shanxi Zhendong Ante Biopharmaceutical Co. Ltd.) administered orally twice daily, 30 min before meals; amoxicillin 1000 mg (Shandong Lukang Pharmaceutical Co. Ltd.) administered orally twice daily, 30 min after meals; and minocycline 100 mg (Hanhui Pharmaceutical Co. Ltd.) administered orally twice daily, 30 min after meals. The treatment regimen for the minocycline dual group was as follows: vonoprazan 20 mg (Takeda Tianjin Pharmaceutical Co. Ltd.) administered orally twice daily, 30 min before meals, and minocycline 100 mg (Hanhui Pharmaceutical Co. Ltd.) administered orally twice daily, 30 min after meals. The treatment duration was 14 days in both groups. Patients were instructed to avoid alcohol for 1 week after completing therapy. In addition, healthcare staff closely monitored and documented any adverse events that occurred during therapy, with particular attention to the most prominent symptoms, which were prospectively assessed and recorded.

The primary outcome was the *H. pylori* eradication rate. Eradication success was assessed 4 weeks after completion of therapy using a 13C urea breath test. Patients received 75 mg of ^13^C‐urea granules orally (Urea [13C] Breath Test Diagnostic Kit; marketing authorization holder: Beijing Huagen Anbang Technology Co. Ltd., China). Breath samples were collected at baseline and 30 min after administration and were analyzed using a 13CO2 detection instrument and the accompanying software. According to the manufacturer's instructions, a detection value of < 4.0‰ was considered negative for *H. pylori* infection and was defined as successful eradication. Patients were instructed to record any adverse events that occurred during treatment and within 3 days after discontinuation of therapy. In the event of serious adverse events, patients were required to contact the study staff promptly for further evaluation and follow‐up documentation. Adverse events were categorized according to their impact on daily life and work as follows: mild: symptoms causing discomfort without affecting daily activities or work; moderate: symptoms causing discomfort with partial impairment of daily activities or work; and severe: symptoms causing substantial impairment of daily activities and work. Treatment adherence was considered good when patients had taken more than 80% of the total prescribed dose.

### 2.3 Statistical Analysis

Statistical analyses were performed using SPSS version 26.0. Continuous variables with a normal distribution were expressed as means ± standard deviations (SDs) and were compared between groups using the independent‐samples *t*‐test. Continuous variables that did not follow a normal distribution were expressed as medians (interquartile ranges [IQRs]) and were compared using the Mann–Whitney *U* test. Categorical variables were expressed as numbers (percentages) and were compared using the *χ* ^2^ test; when the assumptions for the *χ* ^2^ test were not met, the continuity‐corrected *χ* ^2^ test or Fisher's exact test was applied, as appropriate. For the intention‐to‐treat (ITT) analysis, all randomized patients were included, and those lost to follow‐up, those who discontinued treatment because of adverse events, and those who did not complete the prescribed treatment course were considered treatment failures. In the modified intention‐to‐treat (mITT) analysis, patients who did not complete the prescribed treatment course or who discontinued treatment because of adverse events were excluded, whereas patients lost to follow‐up were classified as treatment failures. In the per‐protocol (PP) analysis, patients who were lost to follow‐up, those who discontinued treatment because of adverse events, and those who did not complete the prescribed treatment course were excluded. Between‐group noninferiority was assessed by calculating the two‐sided 95% confidence interval (95% CI) for the treatment difference and performing a one‐sided hypothesis test. A *p* value < 0.05 was considered statistically significant.

### 2.4 Compliance With Ethical Standards

This study was conducted in accordance with the Declaration of Helsinki and applicable ethical standards for clinical research. Before enrollment, all participants were fully informed by the investigators of the study objectives, procedures, potential benefits, and risks, available alternative treatment options, and their right to withdraw at any time. Written informed consent was obtained from all participants voluntarily prior to study entry. During the study, all personal information was deidentified and used exclusively for scientific analysis, with strict safeguards in place to ensure the protection of participant confidentiality.

## 3 Results

### 3.1 Baseline Demographic and Clinical Characteristics of Patients

**FIGURE 1:**
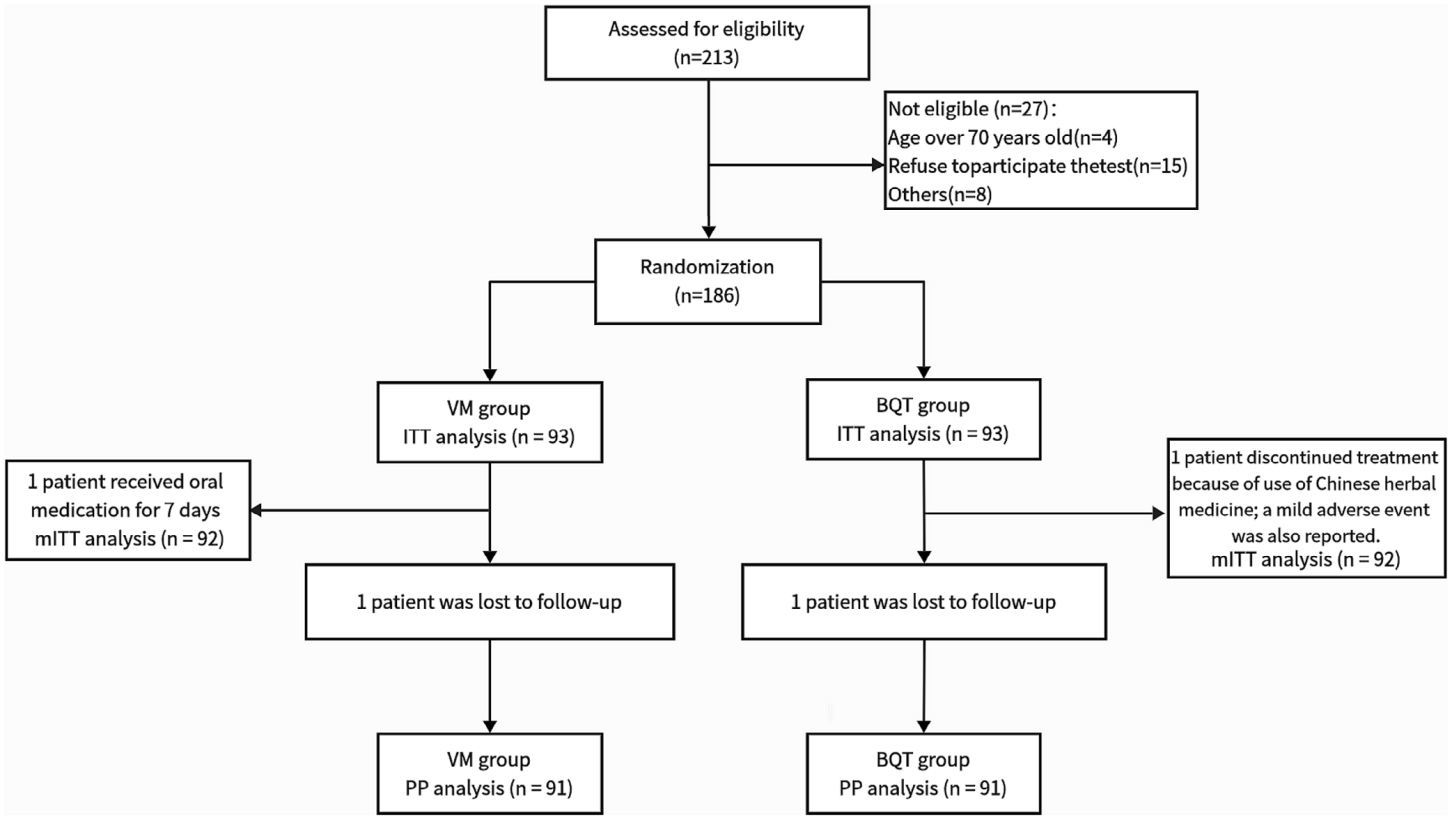
Flowchart of patient enrollment. ITT, intention‐to‐treat; mITT, modified intention‐to‐treat; PP, per‐protocol.

A total of 186 patients were enrolled and included in the ITT analysis, with 93 patients in the bismuth quadruple group and 93 in the minocycline dual group. During treatment, one patient in the bismuth quadruple group failed to complete the prescribed treatment course, and one patient in the minocycline dual group discontinued treatment because of the need to take Chinese herbal medicine for reasons unrelated to the study treatment. This patient also reported a mild adverse event according to the predefined grading criteria; thus, 92 patients in each group were included in the mITT analysis. Subsequently, one patient in each group was lost to follow‐up and was excluded from the PP analysis, leaving 91 patients in each group for the final PP analysis. The study flowchart is presented in Figure 1.

There were no statistically significant between‐group differences in baseline characteristics, including age, sex, body mass index (BMI), smoking history, alcohol consumption, regularity of dietary habits, *H. pylori* infection among household members, consumption of pickled foods, and family history of gastric cancer. Baseline characteristics were well balanced between the bismuth quadruple group and the minocycline dual group (Table 1). The number of previous eradication attempts and the distribution of prior treatment regimen types are summarized in Table 2. No significant between‐group differences were observed in the number of previous eradication attempts or in prior exposure to any of the following regimen types: tetracycline‐based, clarithromycin‐based, levofloxacin‐based, metronidazole‐based, furazolidone‐based, amoxicillin‐based, or bismuth‐containing regimens.

**TABLE 1:** Baseline characteristics of study patients.

Variables	BQT group (*n* = 93)	VM group (*n* = 93)	*p* ^a^
Age (years), median (IQR)	46.00 (34.50–57.50)	49.00 (37.50–57.00)	0.430
BMI, median (IQR)	23.44 (21.80–25.66)	23.83 (21.23–25.66)	0.674
Gender			
Male	42 (45.2)	40 (43.0)	0.768
Female	51 (54.8)	53 (57.0)	
Cigarette smoking	15 (16.1)	16 (17.2)	0.844
Alcohol drinking	20 (21.5)	15 (16.1)	0.348
Irregular dietary habits	28 (30.1)	22 (23.7)	0.321
*H. pylori* infection among family members			
Yes	32 (34.4)	24 (25.8)	0.417
No	31 (33.3)	33 (35.5)	
Not tested	30 (32.3)	36 (38.7)	
Consumption of pickled foods	19 (20.4)	14 (15.1)	0.337
Family history of gastric cancer	10 (10.8)	4 (4.3)	0.095

^a^ The *p*‐values were obtained from two‐sided comparisons of the difference between the BQT group and the VM group.

**TABLE 2:** Previous *H. pylori* eradication history and‐ prior treatment exposure in the two treatment groups.

Variables	BQT group (*n* = 93)	VM group (*n* = 93)	*p* ^a^
Number of previous eradication failures			
1	76 (81.7)	84 (90.3)	0.157
2	10 (10.8)	7 (7.5)	
≥ 3	7 (7.5)	2 (2.2)	
Tetracycline based	5 (5.4)	8 (8.6)	0.567
Clarithromycin based	49 (52.7)	49 (52.7)	1.000
Levofloxacin based	9 (9.7)	9 (9.7)	1.000
Metronidazole based	31 (33.3)	29 (31.2)	0.875
Furazolidone based	5 (5.4)	8 (8.6)	0.567
Amoxicillin based	57 (61.3)	62 (66.7)	0.541
Bismuth based	63 (67.7)	72 (77.4)	0.189

^a^ The *p*‐values were obtained from two‐sided comparisons of the difference between the BQT group and the VM group.

### 3.2 Comparison of Eradication Rates

In the ITT analysis, the eradication rates were 91.4% (85/93; 95% CI, 83.8%–96.2%) in the bismuth quadruple group and 89.2% (83/93; 95% CI, 81.1%–94.7%) in the minocycline dual group, with a between‐group difference of −2.2% (95% CI, −10.5%–6.2%). The difference between the two groups was not statistically significant (*p* = 0.620), whereas the *p* value for the noninferiority test was 0.036. In the mITT analysis, the eradication rates were 92.4% (85/92; 95% CI, 85.1%–96.9%) and 90.2% (83/92; 95% CI, 82.2%–95.4%) in the bismuth quadruple group and the minocycline dual group, respectively, with a between‐group difference of −2.2% (95% CI, −9.7%–5.3%). Similarly, no statistically significant difference was observed between the groups (*p* = 0.601), while the *p* value for the noninferiority test was 0.030. In the per‐protocol (PP) analysis, the eradication rates were 93.4% (85/91; 95% CI, 86.2%–97.5%) in the bismuth quadruple group and 91.2% (83/91; 95% CI, 83.4%–96.1%) in the minocycline dual group, with a between‐group difference of −2.2% (95% CI, −9.4%–5.0%). Likewise, the between‐group difference was not statistically significant (*p* = 0.578), whereas the *p* value for the noninferiority test was 0.024.

Overall, eradication rates were high in both groups across the ITT, mITT, and PP analyses, with no statistically significant between‐group differences observed. Minocycline dual therapy achieved eradication rates comparable to those of bismuth quadruple therapy and met the prespecified criterion for noninferiority in the mITT and PP analyses. However, noninferiority was not conclusively established in the ITT analysis because the lower bound of the 95% CI for the treatment difference (−10.5%) fell slightly below the prespecified noninferiority margin of −10%. The detailed data are shown in Table 3.

**TABLE 3:** *Helicobacter pylori* eradication rates of each group.

Analysis	BQT group	VM group	Difference (95% CI for difference)	*p*‐value for noninferiority^a^	*p* ^b^
ITT	91.4% (83.8%–96.2%) n/*N* = 85/93	89.2% (81.1%–94.7%) *n*/*N* = 83/93	−2.2% (−10.5%–6.2%)	0.036	0.620
MITT	92.4% (85.1%–96.9%) *n*/*N* = 85/92	90.2% (82.2%–95.4%) *n*/*N* = 83/92	−2.2% (−9.7%–5.3%)	0.030	0.601
PP	93.4% (86.2%–97.5%) *n*/*N* = 85/91	91.2% (83.4%–96.1%) *n*/*N* = 83/91	−2.2% (−9.4%–5.0%)	0.024	0.578

Abbreviations: ITT, intention‐to‐treat; MITT, modified intention‐to‐treat; PP, per protocol.

^a^ The *p*‐values were obtained from one‐sided test comparisons of noninferiority between the BQT group and VM group.

^b^ The *p*‐values were obtained from two‐sided comparisons of the difference between the BQT group and VM group.

### 3.3 Comparison of Patient Compliance and Adverse Reactions

Both the bismuth‐containing quadruple therapy and vonoprazan–minocycline dual therapy regimens were well tolerated, with no significant between‐group difference in adherence [97.8% (91/93) vs. 97.8% (91/93), *p* = 1.000]. In the ITT analysis, adverse events were infrequent in both groups, with no significant between‐group differences in the overall incidence or in the frequencies of individual symptoms (all *p* > 0.05). The most common adverse events in the bismuth quadruple group were dizziness, abdominal pain, vomiting, abdominal distension, and diarrhea, whereas those in the minocycline dual group were dizziness, abdominal distension, and abdominal pain. No significant between‐group differences were observed in the incidences of dizziness, nausea, vomiting, acid regurgitation, heartburn, abdominal distension, abdominal pain, diarrhea, or rash (all *p* > 0.05; Table 4). According to the predefined adverse‐event grading criteria, all reported adverse events were mild, and no moderate or severe adverse events were observed in either group.

**TABLE 4:** Rates of adverse events and compliance in each group.

Adverse event and adherence	BQT group (*n* = 93)	VM group (*n* = 93)	*χ* ^2^	*p*
Dizziness	4 (4.3)	4 (4.3)	< 0.001	1.000
Nausea	1 (1.1)	1 (1.1)	< 0.001	1.000
Vomiting	2 (2.2)	0 (0)	2.022	0.155
Acid regurgitation	0 (0)	1 (1.1)	1.005	0.316
Heartburn	0 (0)	1 (1.1)	1.005	0.316
Abdominal distension	2 (2.2)	2 (2.2)	< 0.001	1.000
Abdominal pain	3 (3.2)	2 (2.2)	0.206	0.650
Diarrhea	2 (2.2)	1 (1.1)	0.339	0.561
Rash	0 (0)	1 (1.1)	1.005	0.316
Adverse event	14 (15.1)	11 (11.8)	0.416	0.519
Adherence accoding to AE grade				
Mild	14 (15.1)	11 (11.8)	0.416	0.519
Moderate	0 (0)	0 (0)	—	—
Severe	0 (0)	0 (0)	—	—
Good adherence	91 (97.8)	91 (97.8)	< 0.001	1.000

## 4 Discussion

In this single‐center, prospective, open‐label, randomized, controlled, noninferiority trial, 14‐day minocycline dual therapy achieved eradication efficacy comparable to that of 14‐day bismuth‐containing quadruple therapy as rescue treatment for *Helicobacter pylori* infection. The prespecified noninferiority criterion was met in both the mITT and PP analyses. In the PP population, the eradication rate was 91.2% with minocycline dual therapy and 93.4% with bismuth‐containing quadruple therapy, with an absolute between‐group difference of −2.2% and a 95% confidence interval (CI) of −9.4% to 5.0%. Because the lower bound of the 95% CI remained above the prespecified noninferiority margin of −10%, noninferiority was demonstrated in the PP analysis. However, the ITT result should be interpreted cautiously because the lower bound of the 95% CI slightly crossed the prespecified margin. Taken together, these findings suggest that 14‐day minocycline dual therapy may represent a potential simplified empirical rescue regimen for *H. pylori* infection, particularly when susceptibility testing is not readily available.

In China, particularly in the northwestern region, resistance of *H. pylori* to clarithromycin, metronidazole, and levofloxacin remains high, reaching 55.05%, 85.86%, and 46.46%, respectively [13]. Antimicrobial resistance has therefore become a major determinant of eradication failure. Against this background, failure of first‐line therapy is not uncommon. A randomized, controlled trial reported that empiric clarithromycin‐containing bismuth quadruple therapy achieved an intention‐to‐treat (ITT) eradication rate of 79.4% when used as first‐line treatment, corresponding to a failure rate of approximately 20.6% [14]. These findings underscore the urgent need for effective rescue regimens for patients with first‐line treatment failure. In contrast, resistance rates to amoxicillin and tetracycline‐class antibiotics remain relatively low, at approximately 1%–5% [3], thereby supporting their empirical use in high‐resistance settings. Notably, when combined with potent acid suppression, regimens based on amoxicillin and tetracycline derivatives may achieve satisfactory eradication rates. Previous studies have shown that empiric vonoprazan‐containing rescue regimens generally achieve eradication rates of 80%–90%. In addition, a single‐center real‐world study from China demonstrated that a quadruple regimen consisting of vonoprazan, amoxicillin, minocycline, and bismuth achieved a per‐protocol (PP) eradication rate of 93.7% as second‐line therapy [15]. Collectively, these findings suggest that the combination of potent acid suppression and low‐resistance antibiotics may represent an important strategy for optimizing rescue therapy. Although bismuth‐containing quadruple therapy remains the standard empiric rescue regimen recommended by several consensus statements [2] and was therefore selected as the control regimen in the present study, its substantial medication burden and relatively frequent adverse events may compromise adherence and real‐world effectiveness. By comparison, susceptibility‐guided therapy can improve the precision of rescue treatment, with one study reporting eradication rates of 92.9% in the ITT analysis and 99.0% in the PP analysis [16]. Nevertheless, in regions where access to susceptibility testing is limited and cost remains a concern, empiric therapy continues to be the more feasible and commonly adopted approach. Therefore, identifying an empiric rescue regimen that is simpler, well tolerated, and reliably effective is of substantial clinical importance.

The success of *Helicobacter pylori* eradication is highly dependent on the intensity, consistency, and duration of acid suppression. Current evidence indicates that the efficacy of high‐dose dual therapy (HDDT) is determined not only by the antimicrobial susceptibility of the selected antibiotic(s), but also by the adequacy and durability of acid suppression [17]. In the era of conventional proton pump inhibitors (PPIs), in particular, the efficacy of HDDT was substantially influenced by CYP2C19 genetic polymorphisms, as different metabolic phenotypes were associated with marked variability in intragastric pH control, thereby affecting eradication outcomes. A systematic review by Gao et al. [11], which included 12 randomized, controlled trials involving 2249 patients, showed that PPI–amoxicillin HDDT achieved an intention‐to‐treat (ITT) eradication rate of 83.2% and a per‐protocol (PP) eradication rate of 87.5%, indicating that the regimen did not consistently reach the desired eradication threshold of ≥ 90%. Notably, efficacy declined further in CYP2C19 extensive metabolizers or when acid suppression was inadequate. By contrast, vonoprazan, a potassium‐competitive acid blocker (P‐CAB), provides more rapid, potent, and sustained acid suppression through reversible and competitive blockade of gastric H^+^/K^+^‐ATPase, while being less affected by CYP2C19 polymorphisms [18, 19, 20, 21]. Studies have shown that, under both single‐dose and steady‐state conditions, vonoprazan is superior to conventional PPIs in maintaining an intragastric pH above 4 over a 24‐h period [22]. This more profound and stable high‐pH environment may enhance the intragastric stability of antibiotics and promote a more metabolically active proliferative state of *H. pylori* , thereby improving the efficacy of dual therapy.

Compared with treatment‐naive patients, those undergoing rescue therapy generally have more extensive prior antibiotic exposure, a more complex resistance background, and more heterogeneous causes of prior treatment failure, all of which impose greater demands on regimen design. In this context, the value of a simplified regimen lies not merely in reducing the number of drugs administered, but in its ability to achieve an optimal balance between efficacy and safety through the appropriate selection of low‐resistance antibiotics under sufficiently potent acid suppression. In our previous randomized, controlled study, dual therapy with vonoprazan plus minocycline was shown to achieve an eradication rate of approximately 94% in treatment‐naive patients [23], suggesting that an optimized dual regimen may yield high efficacy when supported by potent acid suppression. In the present study, 14‐day minocycline dual therapy achieved eradication rates comparable to those of bismuth‐containing quadruple therapy and met the prespecified criterion for noninferiority in both the PP and mITT analyses, thereby supporting the feasibility of this regimen as a simplified alternative strategy in the rescue setting. These findings further indicate that the success of dual therapy does not depend on “simplification” per se, but rather on the combination of potent and sustained acid suppression with appropriate antibiotic dosing and administration frequency [24].

Tetracycline‐class antibiotics remain an important option for patients requiring rescue *Helicobacter pylori* eradication therapy. Compared with conventional tetracycline, minocycline offers several notable pharmacokinetic advantages [25, 26]. Its hepatic metabolism and dual routes of elimination (urinary and fecal) reduce reliance on renal excretion alone and may provide more stable drug exposure in patients with fluctuating renal function. Its greater lipophilicity may also enhance tissue penetration and increase drug concentrations within the gastric mucosa. In addition, the oral absorption of minocycline is less affected by food intake, and its relatively long half‐life (approximately 15–23 h) supports twice‐daily dosing. Compared with the conventional four‐times‐daily schedule often required for tetracycline, such a dosing schedule substantially improves treatment convenience and may enhance patient adherence. Accordingly, when a tetracycline‐class agent is indicated, minocycline may represent a more practical therapeutic option.

The minocycline dose used in the present study (100 mg twice daily) was based on dosing regimens commonly used in previous dual‐therapy studies. Notably, the overall eradication rate achieved with this regimen in our study (ITT, 89.2%) was higher than that reported by Gao et al. [10] for minocycline dual therapy as rescue treatment (ITT, 76.7%), suggesting that vonoprazan–minocycline dual therapy may further improve rescue eradication efficacy when supported by potent acid suppression. Importantly, compared with conventional bismuth‐containing quadruple therapy, the minocycline dual regimen used in the present study achieved a similar eradication rate despite reducing exposure to bismuth and an additional antibiotic, and it met the prespecified noninferiority criterion in both the PP and mITT analyses. In a randomized noninferiority trial conducted by Gao et al. in 350 patients with at least one previous eradication failure, the eradication rates were 88.0%, 90.6%, and 91.1% in the ITT, mITT, and PP analyses, respectively [12], findings that are broadly consistent with our results. Taken together, these results suggest that, in the rescue setting—where prior antibiotic exposure is more extensive and resistance patterns are often more complex—a simplified dual regimen based on a low‐resistance tetracycline derivative, when combined with potent acid suppression, may serve as a feasible empirical alternative. It should be noted, however, that patients receiving rescue therapy have, by definition, already experienced at least one previous treatment failure. In this context, the efficacy of a dual regimen consisting of a single antibiotic combined with potent acid suppression may be more vulnerable to interindividual differences in resistance patterns, bacterial burden, and the intragastric environment. The infecting strains in such patients may also have a greater likelihood of antimicrobial resistance or adaptive survival, which may partly explain why the lower bound of the 95% confidence interval in the ITT analysis of the present study marginally crossed the prespecified noninferiority margin. Although the eradication rate of minocycline dual therapy in our study was lower than the eradication rates reported for individualized regimens guided by genotypic resistance testing (ITT, 92.8%; PP, 97.8%) [14], the eradication rates observed in both study groups remained within the range commonly reported for empirical rescue regimens in regions with a high prevalence of antimicrobial resistance (ITT, 75%–90%; PP, 82%–100%) [27, 28].

With regard to safety and tolerability, the present study yielded robust and clinically relevant findings. Minocycline dual therapy was associated with a similarly low overall incidence of adverse events relative to bismuth‐containing quadruple therapy, with no significant between‐group differences in dizziness or gastrointestinal symptoms. Notably, the incidence of dizziness in the minocycline dual group in our study (4.3%) was comparable to that in the bismuth quadruple group and substantially lower than the rate of minocycline‐associated dizziness reported previously (17.9%) [15]. In addition, treatment adherence was high in both groups (> 90%), with no statistically significant between‐group difference, indicating that the 14‐day minocycline dual regimen was well accepted by patients and feasible in clinical practice. This is particularly relevant in the rescue setting, where patients have already experienced prior treatment failure and more complex multidrug regimens may further increase the medication burden and compromise sustained adherence. Beyond short‐term adverse events, the safety of eradication therapy should also be considered in terms of its potential long‐term impact on the gut microbiota. Previous studies have suggested that simplified dual regimens, by reducing exposure to bismuth and additional antibiotics without increasing the overall antibiotic burden, may exert less pronounced effects on the diversity and composition of the gut microbiota than conventional bismuth‐containing quadruple therapy [29].

Several limitations of this study should be acknowledged. First, this was a single‐center, open‐label, randomized, controlled trial with a relatively limited sample size, which may have reduced statistical power, widened the confidence intervals in the noninferiority analysis, and limited the generalizability of the findings. This is particularly relevant to the ITT analysis, in which the lower bound of the 95% CI slightly crossed the prespecified noninferiority margin. Second, the open‐label design may have influenced patient‐reported adverse events and introduced reporting bias in the assessment of tolerability. Third, systematic antibiotic susceptibility testing was not performed. Although regional surveillance data from Ningxia showed low resistance to amoxicillin and tetracycline but high resistance to metronidazole, clarithromycin, and levofloxacin, as well as increased multidrug resistance among patients with previous eradication failure, individual resistance profiles were unavailable in the present trial [30]. Moreover, although previous eradication failures and prior antibiotic or bismuth exposure were generally balanced between groups, these data only reflect potential antibiotic selection pressure and cannot determine actual resistance profiles. Therefore, unknown minocycline resistance or reduced susceptibility may have affected the efficacy of the minocycline dual regimen, while undetected amoxicillin resistance may have influenced the efficacy of the bismuth‐containing quadruple regimen. Unrecognized multidrug resistance may also partly explain heterogeneity in eradication outcomes. Finally, the rescue‐treatment population was heterogeneous with respect to the number of previous failures and prior regimens, and the sample size was insufficient for reliable stratified analyses. Future large‐scale, multicenter, double‐blind, randomized, controlled trials incorporating culture‐based or molecular susceptibility testing are warranted to validate the generalizability of minocycline dual therapy and better define its clinical role.

## 5 Conclusion

In conclusion, 14‐day minocycline dual therapy achieved eradication efficacy comparable to that of 14‐day bismuth‐containing quadruple therapy as rescue treatment for *Helicobacter pylori* infection, with an overall acceptable safety and tolerability profile. The prespecified noninferiority criterion was met in the mITT and PP analyses, whereas noninferiority was not formally established in the ITT analysis. These findings suggest that this simplified dual regimen may serve as a potential empirical rescue option, particularly when susceptibility testing is not readily available and reducing medication burden or treatment complexity is clinically desirable. However, it should not be regarded as a substitute for optimized susceptibility‐guided individualized therapy. Further large‐scale, multicenter, double‐blind, randomized, controlled trials incorporating antibiotic susceptibility assessment are warranted to define its clinical role more precisely.

## Author Contributions

Tingting Yang, Ruijuan Xin, Yanhong Deng, Jun Liu, and Shengjuan Hu made substantial contributions to the conception and design of the study. Tingting Yang, Ruijuan Xin, Yanhong Deng, and Jun Liu were responsible for study implementation, statistical analysis, data interpretation, and drafting and revising the manuscript. Xue Li, Xiaole Guo, Linke Ma, Xiaoming Su, Yanxia Liu, Chengdong Su, Qian Hao, Rui Mu, Xiaofei Li, Pengda Wang, Yanling Li, Xue Zhang, Qingrui Li, Yuting Wu, and Shengjuan Hu made substantial contributions to sample collection, study implementation, data acquisition, data collation, and data monitoring. Shengjuan Hu provided technical and material support, acquired funding, supervised the study, and critically revised the manuscript for important intellectual content. All listed authors made substantial contributions to the manuscript, approved the final submitted version, and agreed to be accountable for all aspects of the work.

## Funding

This work was supported by the Key Science and Technology Achievement Transformation Project of Ningxia Hui Autonomous Region 2024CJE09045.

## Ethics Statement

The study protocol was approved by the Ethics Committee of Ningxia Hui Autonomous Region People's Hospital (Ethics No. [2025]‐LL‐008).

## Conflicts of Interest

The authors declare no conflicts of interest.

## Data Availability

The data that support the findings of this study are available on request from the corresponding author. The data are not publicly available due to privacy or ethical restrictions.
